# NRF2 activation by cysteine as a survival mechanism for triple-negative breast cancer cells

**DOI:** 10.1038/s41388-024-03025-0

**Published:** 2024-04-10

**Authors:** Laura Bottoni, Alberto Minetti, Giulia Realini, Elena Pio, Daniela Giustarini, Ranieri Rossi, Chiara Rocchio, Lorenzo Franci, Laura Salvini, Orazio Catona, Romina D’Aurizio, Mahdi Rasa, Emanuele Giurisato, Francesco Neri, Maurizio Orlandini, Mario Chiariello, Federico Galvagni

**Affiliations:** 1 Department of Biotechnology, Chemistry and Pharmacy, University of Siena, 53100 Siena, Italy; 2 Leibniz Institute on Aging - Fritz Lipmann Institute (FLI), Jena, Germany; 3 Center for Colloid and Surface Science (CSGI), University of Florence, Sesto Fiorentino, 50019 Florence, Italy; 4 Istituto di Fisiologia Clinica (IFC), Consiglio Nazionale delle Ricerche (CNR) and Core Research Laboratory, Istituto per lo Studio, la Prevenzione e la Rete Oncologica (ISPRO), 53100 Siena, Italy; 5 Toscana Life Sciences Foundation, Siena, Italy; 6 Institute of Informatics and Telematics (IIT), CNR, Pisa, Italy; 7 Institute of Immunology, University Medical Center Schleswig-Holstein, Kiel, Germany; 8 Department of Life Sciences and Systems Biology, University of Torino, Torino, Italy; 9 Molecular Biotechnology Center, University of Turin, Torino, Italy

**Keywords:** Transcription, Breast cancer, Stress signalling, Prognostic markers, Mechanisms of disease

## Abstract

Triple-negative breast cancer (TNBC) is a very aggressive and heterogeneous group of tumors. In order to develop effective therapeutic strategies, it is therefore essential to identify the subtype-specific molecular mechanisms underlying disease progression and resistance to chemotherapy. TNBC cells are highly dependent on exogenous cystine, provided by overexpression of the cystine/glutamate antiporter SLC7A11/xCT, to fuel glutathione synthesis and promote an oxidative stress response consistent with their high metabolic demands. Here we show that TNBC cells of the mesenchymal stem-like subtype (MSL) utilize forced cystine uptake to induce activation of the transcription factor NRF2 and promote a glutathione-independent mechanism to defend against oxidative stress. Mechanistically, we demonstrate that NRF2 activation is mediated by direct cysteinylation of the inhibitor KEAP1. Furthermore, we show that cystine-mediated NRF2 activation induces the expression of important genes involved in oxidative stress response, but also in epithelial-to-mesenchymal transition and stem-like phenotype. Remarkably, in survival analysis, four upregulated genes (*OSGIN1*, *RGS17*, *SRXN1*, *AKR1B10*) are negative prognostic markers for TNBC. Finally, expression of exogenous OSGIN1, similarly to expression of exogenous NRF2, can prevent cystine depletion-dependent death of MSL TNBC cells. The results suggest that the cystine/NRF2/OSGIN1 axis is a potential target for effective treatment of MSL TNBCs.

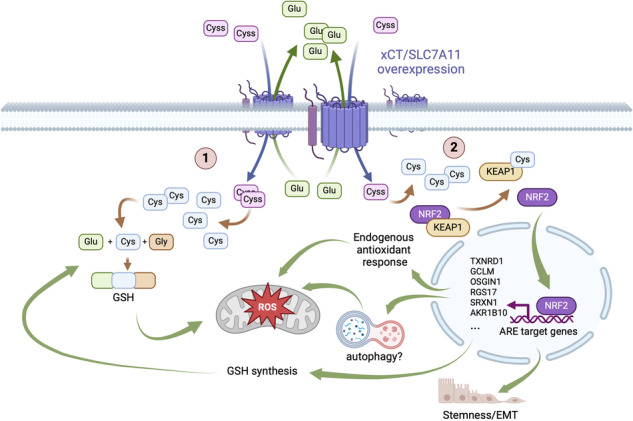

## Introduction

Triple-negative breast cancer (TNBC) is a highly aggressive subtype of breast cancer (BC) that accounts for approximately 15% of all invasive BCs [1]. TNBC is defined by the lack of expression of the biomarkers estrogen receptor (ER), progesterone receptor (PR) and human epidermal growth factor receptor-2 (HER2), which makes TNBC particularly difficult to treat as it is insensitive to the most effective targeted or hormonal therapies currently available [2]. The therapeutic scenario is complicated by the fact that TNBC is a heterogeneous disease and several classifications have been proposed according to specific histological and molecular features [3]. Based on unique gene expression signatures, a classification into seven molecular subtypes has been proposed, namely basal-like 1 (BL1), basal-like 2 (BL2), immunomodulatory (IM), mesenchymal (M), mesenchymal stem-like (MSL), luminal androgen receptor (LAR) and an unclassifiable group (called unstable) [4]. The M and MSL subtypes are characterized by stemness, epithelial-mesenchymal transition (EMT) markers, and an increased potential for invasion and metastasis. Other studies have identified a claudin-low (CL) subtype of TNBC, characterized by low expression of genes involved in tight junctions and epithelial cell-cell adhesion, including claudin 3, 4 and 7, E-cadherin and occludin, and exhibiting chemotherapy-resistant properties [5, 6]. Remarkably, M/MSL and CL subtypes share common features and almost overlap [7, 8]. Such a heterogeneous disease requires a broad spectrum of specific therapeutic options. There is therefore an urgent need to identify the subtype-specific molecular mechanisms underlying TNBC progression and resistance to chemotherapy that may represent therapeutic targets.

The rapid proliferation of tumors requires an increased metabolic rate, which is mainly maintained by mitochondrial respiration, leading to an increased production of reactive oxygen species (ROS) [9]. Above a cytotoxic threshold, ROS-induced oxidative stress (OS) would be detrimental to cancer cells and lead to apoptosis or senescence. Therefore, cancer cells adapt to high ROS concentrations by increasing their antioxidant defense mechanisms.

Nuclear factor erythroid 2-related factor 2 (NRF2), encoded by the NFE2L2 gene, is a transcriptional activator involved in cellular defense against OS. Under normal conditions, NRF2 is degraded by its negative regulator KEAP1 via the ubiquitin-proteasome pathway [10]. However, under electrophilic or oxidative stress, KEAP1 changes its structure and NRF2 dissociates from the complex, accumulates in the nucleus and induces the expression of its target genes involved in the antioxidant response, detoxification, metabolism and inflammation [11]. The NRF2 pathway plays a critical role in the development of chemoresistance and tumor progression in various cancers, and high NRF2 levels in breast cancer and specifically in TNBC are associated with tumor progression and poor prognosis [12–15].

Glutathione (GSH) is the most important molecule among endogenous antioxidants. It acts as a ROS scavenger and plays a crucial role in enzyme-catalyzed antioxidant processes and cellular detoxification against xenobiotics. In cancer, GSH levels are elevated to counteract the increased OS and promote the detoxification of antineoplastic drugs [16, 17]. Consequently, GSH depletion can be used to trigger cancer cell death [18]. GSH is a tripeptide composed of glutamate, cysteine and glycine and is synthesized intracellularly via two reactions catalyzed by the enzymes γ-glutamylcysteine ligase (GCL) and GSH synthetase (GS). In this synthesis process, cysteine (Cys) is the limiting substrate and its cellular uptake as the oxidized form, cystine (Cyss), is mediated by the cystine/glutamate antiporter xCT (encoded by the *SLC7A11* gene). Once inside the cell, Cyss is immediately reduced to Cys, either by oxidation of intracellular GSH or by the NADPH-dependent thioredoxin/thioredoxin reductase 1 system [19]. In TNBC cell lines, xCT/SLC7A11 is overexpressed and its mRNA levels correlate with Cys consumption. Moreover, xCT/SLC7A11 inhibition has a deleterious effect on TNBC cell proliferation, and it is proposed that this effect is mediated by GSH depletion [20].

Here, we present a novel mechanism by which TNBC cells of the MSL/CL subtype activate an OS response through direct induction of the NRF2 pathway by Cyss uptake, and we propose NRF2 and its direct transcriptional target *OSGIN1* as interesting targets for personalized cancer treatment.

## Results

### Cystine deprivation leads to rapid death of TNBC cells by mitochondrial oxidative stress independent of GSH loss

**Fig. 1:**
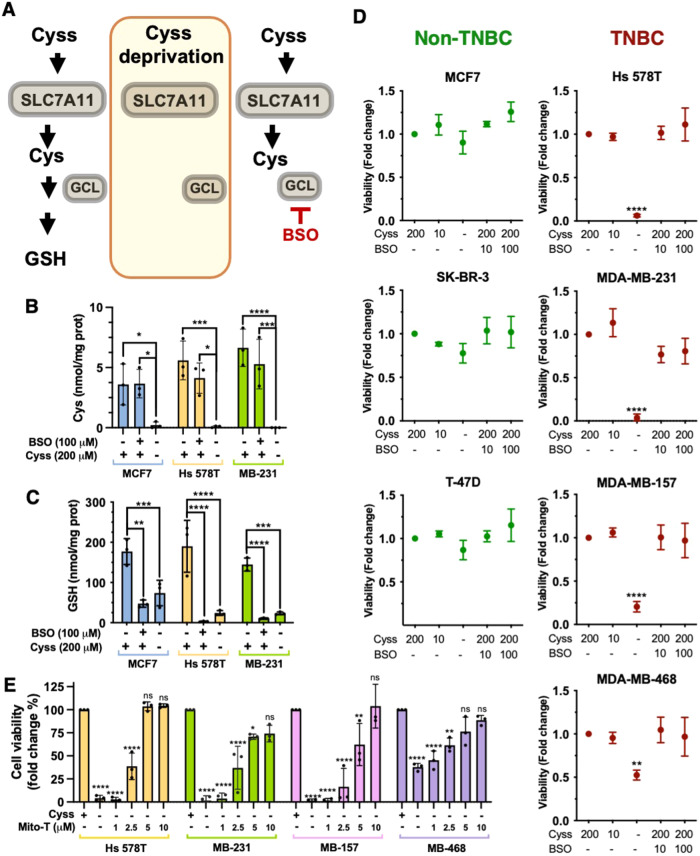
Cystine deprivation leads to rapid death of TNBC cells by mitochondrial oxidative stress, independent of GSH loss. **A** Schematic representation of the uptake of Cyss by the SLC7A11/xCT antiporter and its conversion to GSH (only GCL, the first enzyme in the biosynthetic pathway, is indicated). The effects of Cyss deprivation and BSO treatment on cytoplasmic Cys and GSH content are shown in different font sizes. **B**, **C** Quantitative analysis of cytoplasmic Cys and GSH content after 8 h of Cyss deprivation and BSO treatment. **D** Cell survival of the indicated TNBC and non-TNBC cell lines treated as in (**B**) for 24 h. Cyss and BSO concentrations are reported in μM. **E** Mitochondrial superoxide scavenger mito-TEMPO (Mito-T) rescues cell viability upon cystine deprivation. Data represent the mean ± SD of independent experiments (N = 3). *p ≤ 0.05; **p ≤ 0.01; ***p ≤ 0.001; ****p ≤ 0.0001. ns not significant. One-way (**D**) and two-way (**B**, **C**, **E**) ANOVA were followed by Sidak’s (**B**, **C**), Dunnett’s (**D**), or Tukey’s (**E**) multiple comparisons test.

To investigate the dependence of BC cells on Cyss, we first examined the cytoplasmic Cys/Cyss content in TNBC (Hs 578T and MDA-MB-231) and non-TNBC cell lines (MCF7), cultured for 8 h in the absence of Cyss or in the presence of buthionine sulfoximine (BSO), an inhibitor of GCL (Fig. 1A). In the absence of Cyss, but not in the presence of BSO, cytoplasmic Cys levels were close to zero in all three cell lines tested, while GSH levels were greatly reduced by both Cyss deprivation and BSO treatment (Fig. 1B, C). In the viability assay, Cyss deprivation led to rapid cell death in many TNBC cell lines, but not in non-TNBC cells (Fig. 1D). Conversely, BSO treatment had little or no effect on cell survival, suggesting that GSH depletion does not play a key role in cell death caused by Cys deprivation (Fig. 1D, Fig. S1, and Fig. S2). Finally, since the mitochondrial superoxide scavenger mito-TEMPO restored cell viability, we hypothesized that the observed phenotype was due to mitochondrial oxidative stress (Fig. 1E).

### Cyss deprivation downregulates NRF2 pathway in the MSL subtype of TNBC cells

**Fig. 2:**
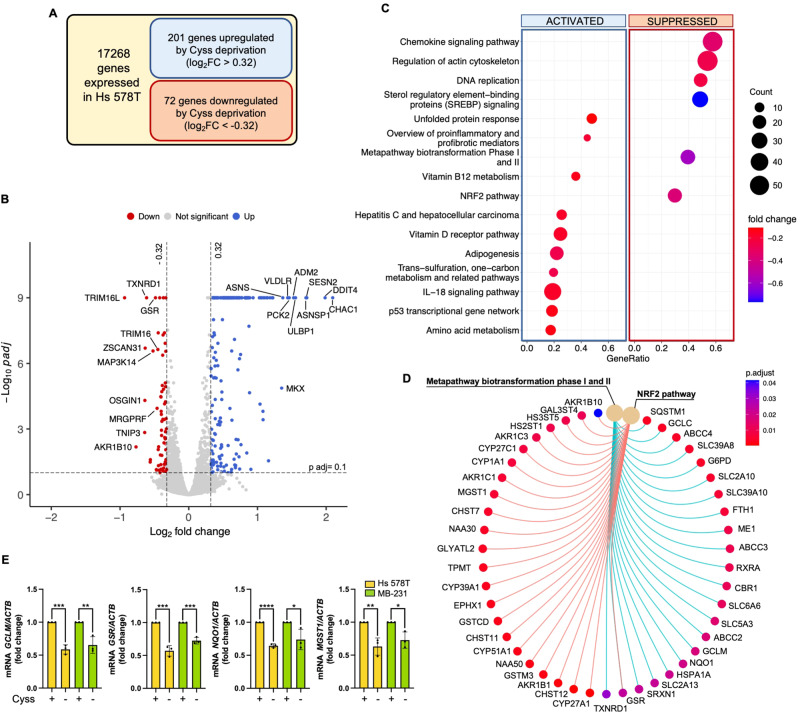
Transcriptome analysis of Hs 578T cells deprived of Cyss from culture medium reveals NRF2 pathway downregulation. **A** Hs 578T cells transduced for the expression of GFP were cultured in complete or Cyss-free medium for 8 h, and gene expression was analyzed by RNA-seq. The transduction with GFP was carried out in anticipation of later experiments with exogenous protein expression. Adjusted p value (p adj) < 0.1 and |log_2_ fold change| (log_2_FC) > 0.32 fold change were used as thresholds for analysis. A gene was considered expressed if there were at least 10 DESeq2 mean normalized counts in either the complete or Cyss-free growth condition. **B** Volcano plot showing the differentially expressed genes (DEGs) after Cyss depletion. Genes down-regulated are shown in red, genes up-regulated are shown in blue. Top ten upregulated and downregulated DEGs are reported. **C** Dot plot showing significantly activated and suppressed Wikipathways (permutation test’s BH p-adj<0.05) resulting from gene set enrichment analysis (GSEA). Dot radii indicate the number of genes annotated with corresponding terms (Count). GeneRatio stands for the relative proportion of involved genes of each pathway (i.e. Count/setSize). **D** Cnetplot plot showing fold changes in expression of genes associated with the “NRF2 pathway” and the closely related “metapathway biotransformation phase I and II”. **E** RT-qPCR analysis of mRNA levels for four known NRF2 transcriptional targets (*GCLM*, *GSR*, *NQO1*, *MGST1*) in the indicated cell lines. mRNA levels are normalized to *β-actin (ACTB)* mRNA levels. Data represent the mean ± SD of independent experiments (N = 3) and are expressed as fold change over control with Cyss in the medium. *p ≤ 0.05; **p ≤ 0.01; ***p ≤ 0.001; ****p ≤ 0.0001. ± Cyss conditions were compared using the two-tailed unpaired *t*-test. An F test was used to compare variance.

Next, we examined the short-term (8 h) effect of Cys deprivation on Hs 578T cells by transcriptome analysis (Fig. 2A, B, and Dataset S1) to investigate the underlying mechanism. Among the significantly modulated pathways, we found downmodulation of the NRF2 pathway and the closely related metapathway biotransformation phase I and II, many genes of which are direct transcriptional targets of NRF2 (Fig. 2C, D, Figs. S3, S4, and Dataset S2). Downmodulation of the NRF2 pathway was also confirmed by RT-qPCR analyses of four known NRF2 transcriptional targets (*GCLM*, *GSR*, *NQO1*, and *MGST1*) in both Hs 578T and MDA-MB-231 TNBC cells, showing reduced expression of these genes upon Cyss deprivation (Fig. 2E).

**Fig. 3:**
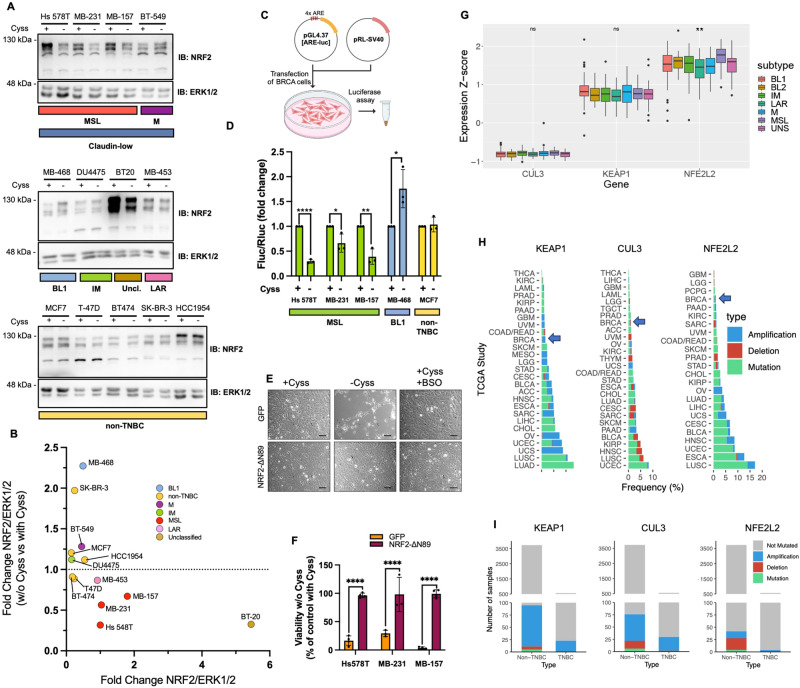
Free Cyss induce NRF2 expression and activity in TNBC cell lines of the mesenchymal stem-like subtype. **A** Western blot analysis of TNBC and non-TNBC cell lines cultured for 16 h in presence or absence of Cyss. TNBC cell lines are assigned to the molecular subtypes according to Lehmann et al. [4]: mesenchymal (M), Mesenchymal stem-like (MSL), basal-like 1 (BL1), Immunomodulatory (IM), luminal androgen receptor (LAR), and unclassified (Uncl.). Analysis of total ERK1/2 expression was used as loading control. **B** Analysis of the band intensity of NRF2. NRF2 levels in cells growing in presence of Cyss are normalized to the ERK1/2 levels, refer to NRF2 expression in Hs 578T cells, and are plotted on the x-axis. On the y-axis, the normalized NRF2 levels obtained in the absence of Cyss are shown for each cell line individually as a fold change compared to the controls with Cyss. Data are expressed as mean values ± SD. **C**, **D** Analysis of NRF2 activity by transient transfection of the indicated breast cancer (BRCA) cells with ARE-*luc2P* reporter plasmid (pGL4.37[ARE-luc]). The Firefly luciferase values were normalized to Renilla luciferase activity (pRL-SV40 vector). Data represent the mean ± SD (*N* = 3) and are expressed as fold change compared to cells grown in presence of Cyss. ± Cyss conditions were compared using the two-tailed unpaired t-test. An F test was used to compare variance. **E** Bright-field images of Hs 578 T cells transduced for the expression of NRF2-ΔN89 or GFP as control and treated for 24 h as indicated. Scale Bar = 100 µm. **F** Viability assay of the indicated cell lines transduced as in (**B**) and grown in presence or absence of Cyss for 24 h. Two-way ANOVA was followed by Tukey’s multiple comparisons test. **G** Boxplot showing *CUL3*, *KEAP1* and *NFE2L2* gene expression distributions among TNBC METABRIC samples grouped by Lehmann subtypes. Significance of Kruskal-Wallis test on median is reported (** for *p* < 0.001). **H** The frequency of samples with altered *KEAP1*, *CUL3* and *NFE2L2* genes in 27 datasets of TCGA collection is shown. The alterations include single nucleotide variants, gene deletion and gene amplification. BRCA: breast cancer. **I** For samples belonging to TCGA BRCA cohort, the number of samples with altered *KEAP1*, *CUL3* or *NFE2L2* is reported, distinguishing between TNBC and non-TNBC. *p ≤ 0.05; **p ≤ 0.01; ****p ≤ 0.0001.

To further investigate these observations and confirm the cystine-dependent modulation of NRF2, we analyzed NRF2 expression in eight TNBC and five non-TNBC cell lines by Western blot. The TNBC cell lines were grouped according to the Lehmann/Pietenpol classification [4, 21]. NRF2 expression was significantly reduced by Cyss depletion in all TNBC cell lines of the MSL subtype and in the unclassified BT-20 (Fig. 3A, B and Fig. S5). Interestingly, all MSL cell lines tested also belong to the CL molecular subtype. Furthermore, NRF2 expression was not decreased by BSO treatment, confirming the viability phenotype observed for BSO-treated cells and demonstrating that the downregulation of NRF2 expression by Cyss depletion is not dependent on GSH (Fig. S6).

To further confirm the downregulation of NRF2 transcriptional activity by Cyss depletion in MSL subtype cells, we transfected a reporter plasmid containing four copies of an antioxidant response element (ARE) that drive transcription of the luciferase gene (Fig. 3C). The results closely mirror what was shown in the Western blots, confirming that Cyss can induce the NRF2 pathway in MSL TNBC cells (Fig. 3D).

Finally, we wondered whether downregulation of the NRF2 pathway is directly responsible for cell death after Cyss depletion. To test this, we expressed a stable NRF2 mutant in Hs 578T, MDA-MB-231 and MDA-MB-157 cells. In this mutant, the first 89 amino acids at the N-terminus (NRF2-ΔN89) were removed, corresponding to the Neh2 domain that mediates NRF2 degradation by binding the repressor KEAP1. Exogenous NRF2 was sufficient to completely prevent loss of cell viability by Cyss depletion, strongly suggesting a role of activated NRF2 in Cyss dependence of the MSL subtype of TNBC cells (Fig. 3E, F). Interestingly, by analyzing the expression of *KEAP1*, *CUL3*, and *NFE2L2* mRNA in TCGA TNBC samples, we found that *NFE2L2* expression is higher in the MSL subtype (Fig. 3G). The Cys-dependent strategy of NRF2 activation adopted by MSL TNBC cells is all the more interesting as breast cancer is one of the cancers with the lowest number of mutations or copy number variations (CNV) in the *KEAP1*, *CUL3* or *NFE2L2* genes that would lead to constitutive activation of this pathway and are more common in other cancers (Fig. 3H, I) [22].

### Cyss uptake by SLC7A11/xCT induces NRF2 expression and activation

**Fig. 4:**
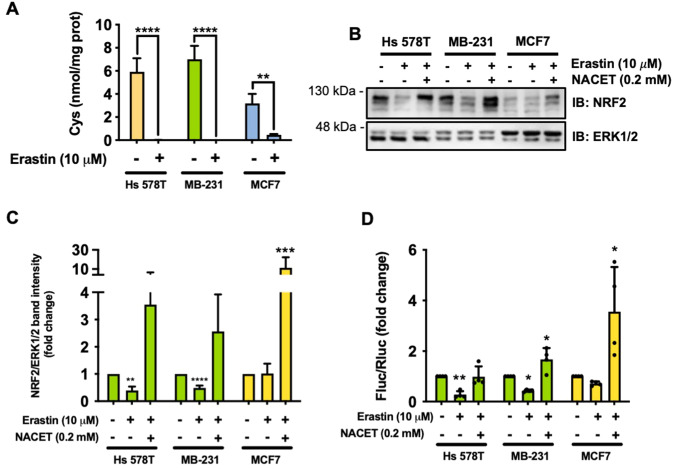
Cyss uptake by SLC7A11/xCT induce the expression and activation of NRF2. **A** Quantitative analysis of cytoplasmic Cys content after 8 h of treatment with Erastin (*N* = 3). **B** Western blot analysis for NRF2 expression in the indicated cell lines treated for 8 h as indicated. **C** Quantification of western blot analyses as in (**B**). Data represent the average ± SD of independent experiments and are expressed as fold change compared to cells grown in absence of Erastin (*N* = 3). **D** Analysis of the NRF2 activity by transient transfection of the indicated cells with ARE-*luc2P* reporter plasmid. Cells were treated as indicated for 8 h. Data represent the mean ± SD (*N* = 4) and are expressed as fold change compared to cells grown in absence of Erastin. *p ≤ 0.05; **p ≤ 0.01; ***p ≤ 0.001; ****p ≤ 0.0001. One-way (**C**, **D**) and two-way (A) ANOVA were followed by Sidak’s (**A**), Tukey’s (**C**), or Dunnett’s (**D**) multiple comparisons test.

Since TNBC cells are known to express high levels of the cystine/glutamate antiporter SLC7A11/xCT, we wondered whether the cellular Cyss uptake and consequently cytoplasmic Cys levels are responsible for the activation of the NRF2 pathway. To investigate this, we first treated Hs 578T, MDA-MB-231 and MCF7 cells with erastin, a potent and selective inhibitor of SLC7A11/xCT [23]. Erastin rapidly deprived the cells of intracellular Cys (Fig. 4A) and decreased NRF2 expression in TNBC but not in non-TNBC MCF7 cells. Treatment of cells with N-acetyl-L-cysteine ethyl ester (NACET), a cell-permeable Cys precursor that does not require active transport [24], restored NRF2 expression (Fig. 4B, C). These data were also confirmed for NRF2 transcriptional activity (Fig. 4D). Taken together, these observations strongly suggest that Cyss uptake induces NRF2 expression and activity in TNBC cells overexpressing SLC7A11/xCT.

### Molecular mechanism of Cyss-regulated NRF2 expression

**Fig. 5:**
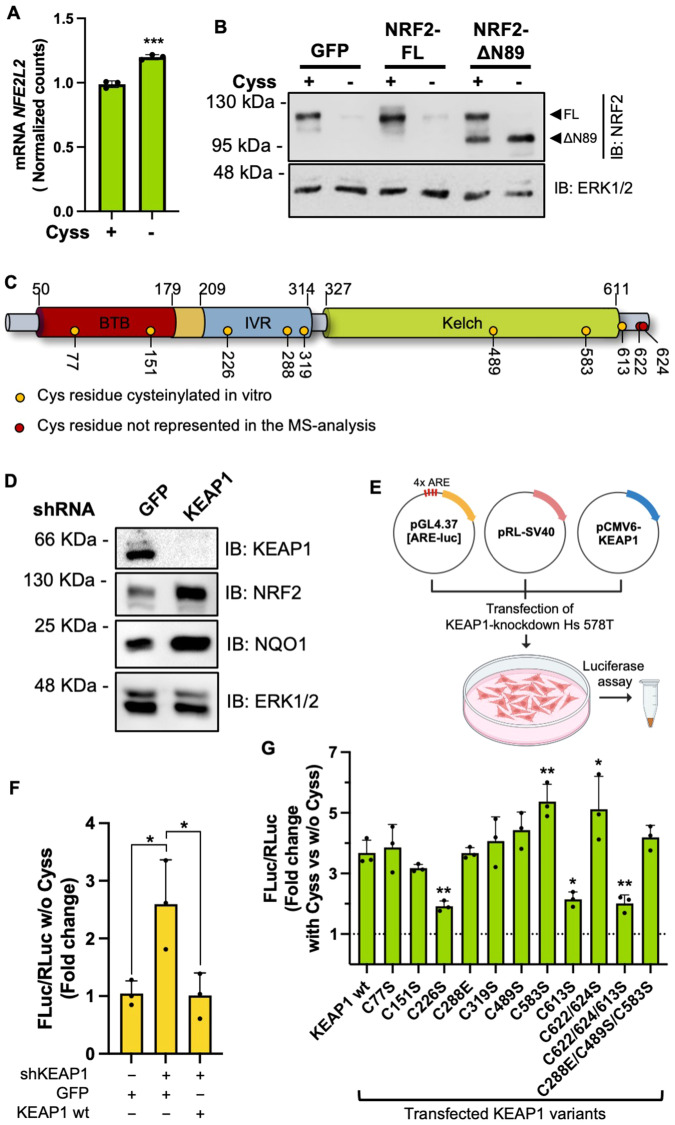
Molecular mechanism of Cyss-regulated NRF2 expression. **A** NRF2 mRNA expression in Hs 578 T cells expressed as normalized counts extrapolated from DESeq2 transcriptome analysis of Fig. 2. ± Cyss conditions were compared using the two-tailed unpaired t-test. An F test was used to compare variance. **B** Western blot analysis of NRF2 expression in Hs 578T cells lentiviral transduced for exogenous expression of NRF2 full length (NRF2-FL) or NRF2-ΔN89. GFP expressing cells were used as negative control. The bands corresponding to NRF2-FL **(**FL) or NRF2-ΔN89 (ΔN89) are indicated by arrowheads. **C** Schematic representation of the domain composition and respective amino acid boundaries for full-length human KEAP1. BTB Broad complex, Tramtrack and Bric-à-Brac, IVR intervening region. The Cys residues identified as cysteinylated by MS analysis are indicated as yellow dots and their position in the polypeptide. **D** Western blot analysis of KEAP1, NRF2 and the NRF2 target NQO1 expression in Hs 578T cells following lentiviral-mediated expression of shRNAs targeting KEAP1 or GFP (as negative control). **E** Scheme of the transfection protocol for the analysis of NRF2 transactivation activity. pRL-SV40 was used for the constitutive expression of Renilla Luciferase for normalization. **F** NRF2 activity measured as transactivation of the ARE-luc reporter gene in cells silenced for endogenous KEAP1 expression (shKEAP1). Cells were transiently transfected with plasmid vectors for the expression of shRNA-resistant wild type KEAP1 or GFP (as negative control). Values are reported as mean ± SD (*N* = 3) and represent the fold change in Renilla-normalized Firefly luciferase activity. **G** as in (**F**) KEAP1 silenced cells were transfected with reporter plasmids and one plasmid for the expression of shRNA-resistant KEAP1 (wt or mutant, as indicated). 24 h post transfection, the cells were grown for 8 h in growth medium ± Cyss. KEAP1 Cys residues were substituted with serine or glutamic acid (C288E) as described by Suzuki et al. [31]. Values represent the fold change in normalized luciferase activity in cells transfected for the expression of the corresponding KEAP1 variant and are expressed as means ± SD (*N* = 3). *p ≤ 0.05; **p ≤ 0.01. One-way ANOVA was followed by Tukey’s (**F**) or Dunnett’s (**G**) multiple comparisons test.

Next, we investigated the molecular mechanism by which Cyss regulates NRF2 expression. Because NRF2 mRNA levels were increased in Hs 578T cells by Cyss depletion (Fig. 5A), we hypothesized that Cyss mediates protein stabilization of NRF2 and that KEAP1 is involved in sensing intracellular Cyss levels by direct cysteinylation of its sensor Cys residues. To test this, we first examined the effect of Cyss deprivation on the expression of exogenous full-length NRF2 (NRF2-FL) and NRF2-ΔN89 and found that only the NRF2-ΔN89 deletion mutant is insensitive to Cyss deprivation, confirming the involvement of KEAP1 in this regulation (Fig. 5B). To identify the sensor Cys residues of KEAP1 involved in direct cysteinylation by free Cys, we incubated the recombinant purified KEAP1 with free Cys and determined by mass spectrometry that Cys residues 77, 151, 226, 288, 319, 489, 583, and 613 were modified by cysteinylation in vitro (Fig. 5C, Table S1). Because the Cys residues of KEAP1 can be oxidized in vitro in a non-specific manner [25], we used these preliminary results to confirm the sensor residues in a functional assay in the cell environment. To this end, we first knocked down the expression of KEAP1 in Hs 578T cells by shRNA targeting the 3′UTR of KEAP1 mRNA (Fig. 5D). We then transfected the silenced cells and the control cells with plasmids for expression of the ARE-luc reporter gene and the ORF of KEAP1 (wt or point mutated in the identified sensor residues), which lacks its 3′UTR sequence and is therefore resistant to the shRNA used (Fig. 5E). Cys residues 622 and 624 were also included in this analysis because peptides containing these C-term residues were not detected in the MS analysis and because they have been described to be involved in KEAP1 inactivation in synergy with residues 226 and 613 [25]. As expected, silencing of endogenous KEAP1 increased luciferase activity driven by ARE elements under Cyss depletion conditions, whereas simultaneous expression of exogenous shRNA-resistant KEAP1 restored basal expression (Fig. 5F). Finally, in mutant analysis, only mutations of Cys residues 226 and 613 resulted in defective induction of ARE activity in the presence of Cyss (Fig. 5G). Since Cys is degraded to serine and H_2_S in the cell and H_2_S induces S-sulfhydration of KEAP1 and stabilization of NRF2 (ref. [26]), we wondered if Cys could be responsible for NRF2 expression through H_2_S production. To test this, we treated Hs 578T cells with amino-oxyacetic acid (AOAA) and I3MT-3, pharmacological inhibitors of endogenous H_2_S-producing enzymes, and observed no decrease in NRF2 expression (Fig. S7). Taken together, these results suggest that in TNBC cells cytoplasmic Cys is involved in the post-translational stabilization of NRF2 by direct cysteinylation of residues 226 and 613 of KEAP1 and suppression of its activity.

### NRF2 target genes downstream of its Cyss-dependent activation

**Fig. 6:**
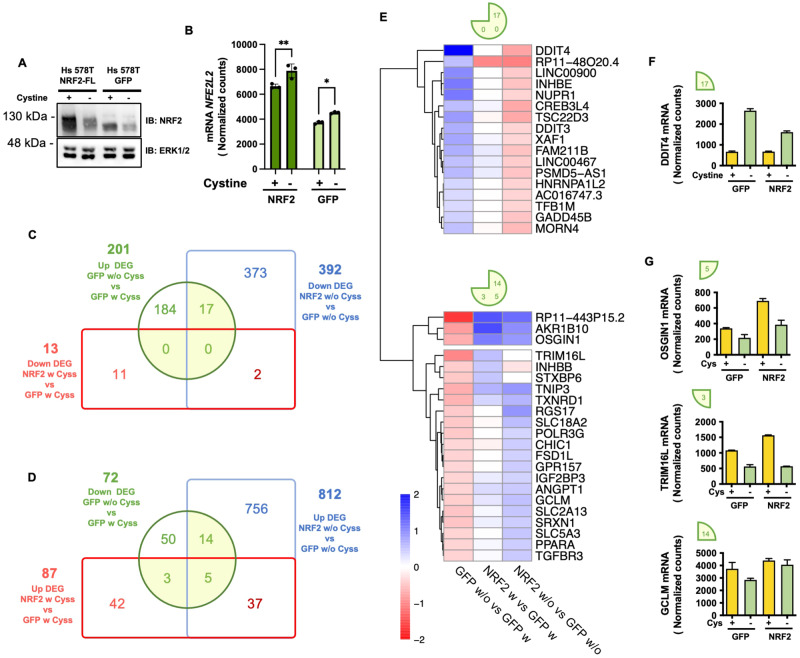
NRF2 target genes downstream of its Cys-dependent activation. **A** Western blot analysis of NRF2 expression in Hs 578T cells transduced with lentiviral vectors for the expression of NRF2-FL (NRF2) or GFP (as control). Whole protein extracts were prepared after 8 h of growth in presence (w Cyss) or absence (w/o Cyss) of Cyss. The same cells as in (**A**) were processed for RNA-seq analysis. **B** DESeq2 mean of normalized counts for NRF2 gene (NFE2L2). Two-way ANOVA was followed by Sidak’s multiple comparisons test. **C** Euler diagram showing effect of the expression of exogenous NRF2 on DEGs upregulated in GFP control cells by Cyss depletion. **D** Euler diagram showing the effect of exogenous NRF2 expression on downregulated DEGs in GFP control cells by Cyss depletion. Adjusted *p* value (p adj) <0.1 and |log_2_FC| > 0.32 were used as thresholds for analysis. **E** Hierarchical clustering of selected DEGs in control cells following Cyss (GFP w/o Cyss vs GFP w Cyss, |log_2_FC| > 0.32, p-adj > 0.1), considering their expression fold change relative to all the compared conditions. Color scale refers to the fold change values of DE transcripts. **F**, **G** DESeq2 mean normalized counts for one gene from each subgroup of NRF2-modulated genes as example. The corresponding pie chart segments are drawn.

Next, we wanted to identify NRF2 target genes that are modulated by Cyss. To this end, we expressed exogenous NRF2-FL in Hs578 cells and analyzed the transcriptome after 8 h of Cyss deprivation in comparison to cells grown in the presence of Cyss and to cells expressing GFP as a control and treated in the same manner (Fig. 6A, B and Datasets S1, S3–6). This analysis identified 17 genes that were upregulated in control cells after Cyss deprivation and significantly downregulated by NRF2 in the absence of Cyss (Fig. 6C, E, F), and 22 genes that were downregulated in control cells and significantly upregulated by NRF2 in the presence of Cyss (3 genes: *STXBP6*, *INHBB*, and *TRIM16L*), in the absence of Cyss (14 genes: *SLC5A3*, *ANGPT1*, *GCLM*, *PPARA*, *IGF2BP3*, *TGFBR3*, *POLR3G*, *GPR157*, *CHIC1*, *SLC18A2*, *FSD1L*, *SRXN1*, SLC2A13, and *RGS17*) or in both conditions (5 genes: *TXNRD1*, *RP11-443P15.2*, *AKR1B10*, *OSGIN1*, and *TNIP3*) compared to control cells treated in the same way (Fig. 6D, E, G).

**Fig. 7:**
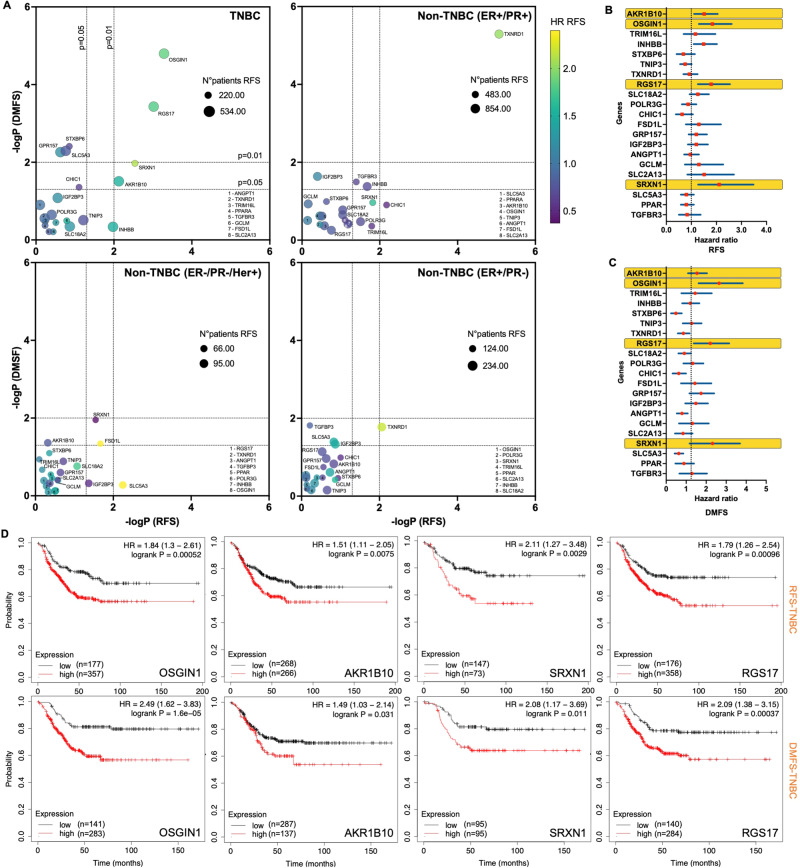
The prognostic values of upregulated genes downstream of NRF2 activation by Cyss in TNBC and non-TNBC. **A** The Kaplan-Meier plotter database was used to analyzed RFS and DMFS for TNBC and non-TNBC patients with high or low expression of the indicated genes. Bubble plot reports -log_10_ of the p values (-logP), the hazard ratio (HR) of RFS and the number of patients for each analysis. **B**, **C** Forest plot reporting the HRs and the 95% confidential intervals of RFS (**B**) and DMFS (**C**) for 21 genes downregulated by Cyss depletion and upregulated by NRF2 (RP11-443P15.2 was not reported because absent in the database). **D** Kaplan-Meier plots of the indicated genes for TNBC patients.

**Fig. 8:**
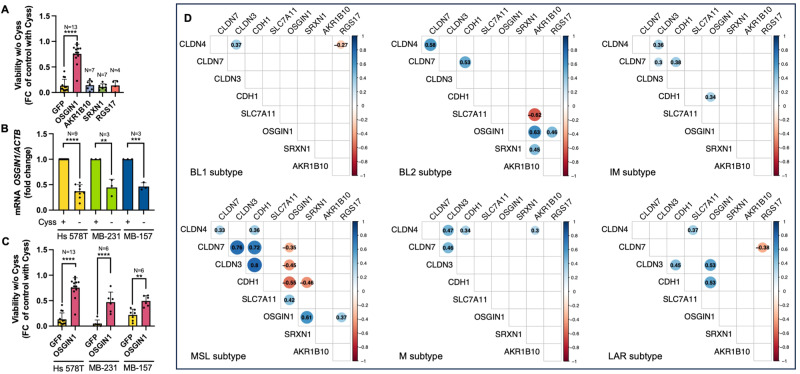
*OSGIN1* rescues Cyss depletion-induced cell death in MSL TNBC cells and correlates positively with *SLC7A11* expression and negatively with *CLDN3*, *CLDN7* and *CDH1* expression in the MSL subtype of TNBC. **A** Cell viability assay of Hs 578 T cells transduced for the exogenous expression of OSGIN1, AKR1B10, SRXN1, RGS17, and GFP as negative control. Values represent the fold change in viability of cells incubated for 24 h without Cyss compared to the same cells incubated with Cyss. One-way ANOVA was followed by Dunnett’s (G) multiple comparisons test. **B** RT-qPCR analysis of the *OSGIN1* expression in MSL TNBC cells in presence or absence of Cyss. ± Cyss conditions were compared using the two-tailed unpaired t-test. An F test was used to compare variance. **C** Cell viability assay of Hs 578 T, MDA-MB-231, and MDA-MB-157 cells transduced for the exogenous expression of OSGIN1, and GFP as negative control. Values represent the fold change in viability of cells incubated for 24 h without Cyss compared to cells incubated with Cyss. Two-way ANOVA was followed by Sidak’s multiple comparisons test. Values of (**A**), (**B**) and (**C**) are expressed as mean ± SD. **p ≤ 0.01; ***p ≤ 0.001; ****p ≤ 0.0001. **D** Correlograms with pairwise Spearman correlation of gene expression levels. Positive correlations are shown in blue, while negative correlations are shown in red. The intensity of the colors is related to correlation coefficients (R). Each correlogram is specific for a Lehman subtype and only significant correlations (Spearman rho correlation test’s *p* < 0.05) were reported.

To evaluate the impact of these NRF2 target genes on TNBC prognosis, we plotted the significance (−log_10_ of p-values) of logrank tests for DEGs downregulated by Cyss depletion using the Kaplan-Meier plotter platform and based on relapse-free survival **(**RFS) and distant metastasis-free survival (DMFS) as endpoints of patients with TNBC and ER+/PR+, ER+/PR−, and ER−/PR−/Her+ non-TNBC [27]. In TNBC patients, mRNA expression levels of *OSGIN1, AKR1B10, SRXN1* and *RGS17* were positively correlated with both worse RFS (hazard ratios 1.84, 1.51, 2.11, and 1.79, respectively) and with a worse DMFS (hazard ratios 2.49, 1.49, 2.08, and 2.09, respectively) (Fig. 7A–D). In non-TNBC patients,of these four genes, only *SRXN1* showed a positive correlation with worse RFS in ER+/PR+ patients (Fig. 7A and Fig. S8). We then investigated whether some of these genes also play a key role in NRF2-mediated survival of TNBC cells in the absence of Cyss. To this end, we exogenously expressed *OSGIN1*, *RGS17*, *SRXN1* and *AKR1B10* in Hs 578T cells and analyzed viability. Under these experimental conditions, only *OSGIN1* was able to almost completely reverse the loss of viability (Fig. 8A). Remarkably, *OSGIN1* was similarly regulated by Cyss (Fig. 8B) and promoted survival in the absence of Cyss (Fig. 8C) in the other MSL cells as well.

To support the hypothesis that Cyss uptake underlies the expression of these four NRF2 target genes in MSL/CL TNBC, we next examined the correlation of their expression with the expression levels of the *SLC7A11*, E-cadherin (*CDH1*) claudin 3 (*CLDN3*), and claudin 7 (*CLDN7*) genes in TNBC patients (*n* = 280) of METABRIC cohort [28]. Patients were stratified into the six Lehmann subtypes to analyze the association. In the MSL subtype, we observe a positive correlation between *OSGIN1* and *SLC7A11* expression (*R* = 0.42; *p* = 0.01) and a negative correlation between *OSGIN1* and *CLDN3* (*R* = −0.45; *p* = 0.06), *CLDN7* (*R* = −0.35; *p* = 0.03), and *CDH1* (*R* = −0.55; *p* = 0.0005) (Fig. 8D).

## Discussion

TNBC cells overexpress *SLC7A11*, which is thought to promote cell survival under OS conditions by sustaining a high rate of GSH synthesis [20]. This is confirmed by the sensitivity of these cells to BSO and by the direct correlation between BSO sensitivity and *SLC7A11* expression [20, 29]. Here, we confirm the Cys dependence of TNBC cells, but by comparing Cyss deprivation with BSO treatment, we show that among the BC cell lines examined, TNBC cells of the MSL/CL subtype exhibit the strongest Cys dependence and in a GSH-independent manner. Indeed, BSO-induced cell death is achieved when cells are treated for 72 h or longer [29], whereas 24 h is sufficient to achieve cell death by Cyss deprivation. These results suggest that there is a pathway downstream of Cyss uptake, but independent of GSH synthesis, that can support OS resistance. In this regard, we demonstrated that NRF2 expression and signaling is upregulated by Cyss uptake in the cell lines with the highest *SLC7A11* expression belonging to the MSL/CL subtype and the unstable group (BT-20). It is worth noting that the BT-20 cell line was recently reclassified as MSL [30]. Overall, these observations, combined with the results of mass spectrometry and KEAP1 mutant analysis and the fact that TNBCs are characterized by a truly low rate of somatic mutations and CNV of *NFE2L2* and genes encoding key components of the NRF2 degradation pathway, are consistent with the hypothesis that forced Cyss uptake by overexpression of *SLC7A11* induces KEAP1 inactivation via cysteinylation of sensor residues 226 and 613. Therefore, Cyss can be considered a new member of class IV of inducers that activate NRF2 signaling independently of Cys151/Cys273/Cys288 [31]. Interestingly, it was previously proposed that the ability of KEAP1 to sense H_2_O_2_ depends on the formation of a disulfide bond between Cys 226 and 613, and this sensing mechanism differs from that used by electrophilic NRF2 inducers, suggesting the plausibility of cysteinylation of these two residues in cells [25]. However, in the absence of evidence for cysteinylation of KEAP1 in cells, we cannot completely exclude the possibility that Cys signaling is mediated indirectly.

By transcriptome analysis of Hs 578T cells, we identified NRF2 target genes downstream of Cyss uptake. The DEGs with known function that are positively regulated by Cyss deprivation in an NRF2-dependent manner are stress response genes involved in endoplasmic reticulum (ER) stress response and autophagy (*DDIT4*, *CREB3L4*, *DDIT3*, *XAF1*, NUPR1, and *GADD45B*) [32–36], mitophagy (*MORN4*) [37] or mitochondrial biogenesis (*TFB1M*) [38]. Since NRF2 is a transcriptional activator, we hypothesize that these genes are rapidly activated by OS and ER stress induced by Cyss deprivation and are only indirectly regulated by NRF2 as it plays a role in balancing OS, mitochondrial dysfunction and also ER stress [39, 40] Of the 22 DEGs that were negatively regulated by Cyss deprivation and positively regulated by exogenously expressed NRF2, 19 remained upregulated in the presence of exogenously expressed NRF2 even in the absence of Cyss and consequently could be direct targets of NRF2 transcriptional activity and involved in the survival of Hs 578T cells under these conditions. In support of this hypothesis, six of these DEGs (*TNXRD1*, *SRXN1, GCLM, AKR1B10, OSGIN1, and TRIM16L)* are already known to be direct NRF2 transcriptional targets [41–46]. Among these 19 DEGs, many play a role in TNBC or BC in general and are worth mentioning. *GCLM*, *SRXN1* and *TXNRD1* are directly involved in GSH- or NADPH-dependent antioxidant pathways [47]. *AKR1B10* encodes a multifunctional NADPH-dependent reductase that stimulates BT-20 breast cancer cell growth in vitro and metastasis in vivo [48, 49]. *RGS17* is upregulated in BC where promotes cell migration, invasion, and proliferation [50]. *IGF2BP3* is preferentially expressed in TNBC, where it is significantly more abundant in tumor-initiating BC cells and promotes EMT and stem-like properties [51–54]. *TGFBR3* is specifically overexpressed in MSL TNBC and has been shown to act as a tumor promoter for MSL TNBC cells [55]. *POLR3G* is a critical driver of stemness [55] and is significantly overexpressed in TNBC but not in other breast cancers [56]. In addition, *POLR3G* promotes invasive growth and metastasis of MDA-MB-231 cells [56]. Overall, these observations suggest that Cyss uptake in TNBC can maintain NRF2 activation to promote substantial OS resistance, but also, less obviously, EMT and stem-like phenotype of MSL TNBC. This is also consistent with the emerging role of NRF2 in promoting stem cell properties in cancer cells [57].

Finally, we found that the expression of exogenous OSGIN1, similarly to the expression of exogenous NRF2, can rescue Cyss depletion-induced rapid cell death in MSL/CL TNBC cells, suggesting for the first time a role of *OSGIN1* as a tumor promoter. *OSGIN1* can promote apoptosis [58, 59] or autophagy [60] and cytoprotection [61]. Based on the observations that in TNBC a high level of autophagy contributes to the maintenance of cellular homeostasis and promotes tumor progression and chemotherapy resistance, we speculate that OSGIN1 may play a protective role in stress-induced cell death in MSL/CL TNBC cells by promoting autophagy. However, this hypothesis needs further and more detailed investigation.

In conclusion, TNBC is an aggressive and heterogeneous tumor that requires more selective treatment, and the xCT/NRF2/*OSGIN1* axis is a novel specific target for the treatment of MSL/CL subtypes overexpressing *SLC7A11*, which may be more effective than GSH depletion.

## Materials and methods

### Cell culture and treatment

Cells were cultured as described in Table S2. For Cyss depletion, cells were washed with phosphate-buffered saline solution (PBS), pH 7.4, and incubated for the indicated times in DMEM, high glucose, no glutamine, no methionine, no cystine (Gibco, Thermo Fisher Scientific, Waltham, MA, USA) supplemented with 10% (v/v) fetal bovine serum (FBS), 0.2 mM l-Methionine, 100 μg/ml streptomycin, 100 U/ml penicillin, and 2 mM l-glutamine. 0.8 or 0.2 mM l-Cystine was added in control cells. Lenti-X 293T cell line was used to produce lentiviral particles. For cells treatments, AOAA (Selleck Chemicals LLC, Houston TX 77230 USA), I3MT-3 (MedChemExpress, Sollentuna, Sweden**)**, and Erastin **(**Tocris-Bio-Techne SRL, Milano, Italy) were solubilized in DMSO. BSO (Sigma–Aldrich, Milan, Italy) and NACET [24] were solubilized in water. Mito-TEMPO (Sigma-Aldrich, Milan, Italy) was solubilized in DMSO and added to the cells simultaneously with the medium change 24 h before the viability assay. Cell viability was evaluated by using the CellTiter 96® Aqueous one-solution cell proliferation kit (Promega, Fitchburg, WI, USA). Intracellular levels of Cys and GSH were measured as previously described [62].

### RNA extraction, RT-qPCR and RNA-Seq

Total RNA was extracted from Hs 578 T cells using RNeasy Plus Mini Kit (Qiagen, Hilden, Germany), according to the manufacturer’s instruction. Total RNAs used to evaluate the gene expression by means of QuantiNova SYBR Green RT-PCR Kit Kit and the Rotor-Gene Q thermocycler (Qiagen, Hilden, Germany). Primers for RT-qPCR are reported Table S3. For gene expression profiling, total RNA of GFP or NRF2 expressing Hs 578 T cells was purified using EuroGOLD TriFast™ (Euroclone, Milan, Italy). RNA quantity and quality were evaluated by Nanodrop 8000 (Thermo Fisher Scientific, Waltham, MA, USA) and Fragment Analyzer (Advanced Analytical Technologies, Heidelberg, Germany) using the DNF-471 Standard Sensitivity RNA Analysis Kit (15nt) (Agilent Technologies, Santa Clara, CA, USA). RNA samples were further processed for mRNA-seq library preparation according to the manufacturer’s instructions (TruSeq RNA Sample preparation v2, Illumina, San Diego, CA, USA). The sequencing was done using an Illumina NextSeq 500 sequencer (single end). Fastq files quality check was performed using FastQC v0.11.5. The fastq files were mapped to the hg19 genome using TopHat v2.1.0 with the following parameters –bowtie1 –no-coverage-search -a 5. The number of reads covered by each gene was calculated by HTSeq-Count 0.11.2 with -s no -a 0 -t exon -m intersection-nonempty parameters. Before further analysis, all of reads mapped to rRNA were removed from the count data. For calculating differentially expressed genes and normalized count, DESeq2 R package v1.20.0 was used with the default parameters. Adjusted p-value < 0.05 calculated by DESeq2 was used to define differentially expressed genes. Gene Set Enrichment Analysis (GSEA), as implemented in the R/Bioconductor package clusterProfiler (v 4.4.4), was used to search for modulated pathways of WikiPathways database (https://www.wikipathways.org/). Mutation profiles, comprising SNV and CNV (amplifications and homozygous deletions) and their frequencies, for the *KEAP1*, NRF2 and *CUL3* genes were downloaded from cBioportal for all 28 studies forming the TCGA PanCancer dataset.

### TNBC subtyping

Expression profiles of TNBC samples (*n* = 320) from the METABRIC dataset were quantile normalized and standardized. After checking the ER expression level, 4 samples were excluded. We used the classifier developed by Chen et al. [63] (https://cbc.app.vumc.org/tnbc/).

### Western blot analysis

Total cell extracts were prepared as previously described [64]. Primary and secondary antibodies used for immunoblotting are reported in Table S4. Band intensities were measured by means of ImageQuant™ LAS 4000 Chemiluminescence Camera System and ImageQuant^TM^ TL analysis software (GE Healthcare Life Science, Chicago, IL, USA).

### Cell transfection and luciferase reporter assay

Cells were seeded to reach 70–80% of confluency on the day of transfection in a 6-well culture plate. 24 h post seeding, 1.8 μg of luciferase reporter vector pGL4.37 (*luc2P*/ARE/Hygro) (Promega Corp., Fitchburg, WI, USA), 0.2 μg of Renilla luciferase control vector (pRL-SV40) (Promega Corp., Fitchburg, WI, USA) were used for the transfection of a single well with the Transporter 5 Transfection Reagent (PolyScience, Niles, IL, USA). For KEAP1 mutant analysis, 1.6 μg of pGL4.37, 0.2 μg of KEAP1 or GFP expression vector, and 0.2 μg of pRL-SV40 were used for the transfection of a single well. 24 h post transfection, the cells were grown in medium with or without Cyss. After 8 h, cells were harvested in Passive Lysis Buffer (Promega Corp., Madison, WI) and the luciferase activity was measured by the Nano Dual-luciferase report™ assay System (Promega Corp., Madison, WI). Luciferase activity was normalized to the activity of the Renilla luciferase as an internal control.

#### Lentiviral transduction

Lentiviral particles were produced by transfecting Lenti-X 293T cell line as previously described [65]. For transduction, the cells were plated in 6-well plates at a concentration of 2.5 × 10^5^ cells·well^−1^. After 24 h, the culture medium was replaced with complete medium and 0.025–0.250 ml of Lenti-X 293 T supernatant containing lentiviral particles and 4 μg/ml of polybrene (hexadimethrine bromide, Sigma-Aldrich, Milan, Italy), per well. After 24 h, medium was changed, and after other 48 h, cell extracts were analyzed by western blot or the total RNA was collected.

### Statistical analysis

The data analysis was performed using Prism 9 statistical software (GraphPad Software Inc., San Diego, CA). Shapiro–Wilk test was used to test for non-Gaussian distribution. Significance was estimated by one-way, two-way ANOVA, or two-tailed unpaired t-test as appropriate to the experimental design. After the ANOVAs were performed, a Sidak’s, Dunnett’s, or Tukey’s multiple comparisons test was used to compare the means of the different groups.

### Plasmids and site-directed mutagenesis

For the RNA interference-mediated knockdown of KEAP1, we used the lentiviral plasmid pLKO.1 from the TRC shRNA library (Sigma-Aldrich, Milan, Italy) expressing specific shRNA for 3′UTR of human KEAP1 (#TRCN0000155340) or GFP as negative control [65]. Lentiviral plasmid for KEAP1 expression (#RC202189L3) and NRF2 (#RC204140L1) were purchased from ORIGENE (Rockville, MD, USA). Lentiviral plasmid for SLC7A11 expression (#118702) expression was purchased from Addgene (Cambridge, MA, USA). The point mutations in human KEAP1 cDNA were introduced using the QuickChange XL Site-Directed Mutagenesis kit (Agilent, Santa Clara, CA).

### KEAP1 mass spectrometry analysis

For mass spectrometry analysis, 10 μg of recombinant human KEAP1 (#11981-H29B-100, Sino Biologicals, Beijin, PR China) were diluted to 100 μl with 20 mM Tris pH 8.0 and diafiltrated by means of a Amicon Ultra-0.5 ml device (Sigma-Aldrich, Milan, Italy) to eliminate GSH, and then treated with 30 mM M DTT and incubated 30 min at room temperature to reduce the disulfide bridges, diafiltrated to remove the excess of DTT and diluted in 20 mM Tris pH 8.0. The reaction volume was divided in 4 aliquots (45 μl; 1 μM final concentration of KEAP1), and to each aliquot were added 5 μl of water or Cys at the concentration of 10 μM. The reaction was incubated 1 h at room temperature, diluted with 20 mM Tris pH 8.0, diafiltrated, rediluted and the protein digested by trypsinization for 3 h. The analyses were performed on a Q-Exactive Plus mass spectrometer. The raw data obtained were analyzed using the Biopharma Finder 2.1 software (Thermo Fisher Scientific).

### Supplementary information

Dataset 1

Dataset 2

Dataset 3

Dataset 4

Dataset 5

Dataset 6

Supplementary material

## Data Availability

The data generated in this study are available within the article and its supplementary information files. The raw data generated in this study are publicly available in Gene Expression Omnibus (GEO) at GSE251707 (Token: ulcrwqyorruftsz).
