# Supplementary material for: NRF2 activation by cysteine as a survival mechanism for triple-negative breast cancer cells
Source: Oncogene. 2024 Apr 10;43(22):1701–13. doi: 10.1038/s41388-024-03025-0 (PMC11136656; doi:10.1038/s41388-024-03025-0)
Supplement: Supplementary file 7 — Supplementary material [file 41388_2024_3025_MOESM7_ESM.pdf]

NRF2 activation by cysteine as a survival mechanism for triple-negative breast cancer cells

Laura Bottoni, Alberto Minetti, Giulia Realini, Elena Pio, Daniela Giustarini, Ranieri Rossi, Chiara Rocchio, Lorenzo Franci, Laura Salvini, Orazio Catona, Romina D'Aurizio, Mahdi Rasa, Emanuele Giurisato, Francesco Neri, Maurizio Orlandini, Mario Chiariello, Federico Galvagni

Supplementary material

Supplementary figures: Figures S1-S8
Supplementary Tables: Tables S1-S4
Supplementary methods

Supplementary figures

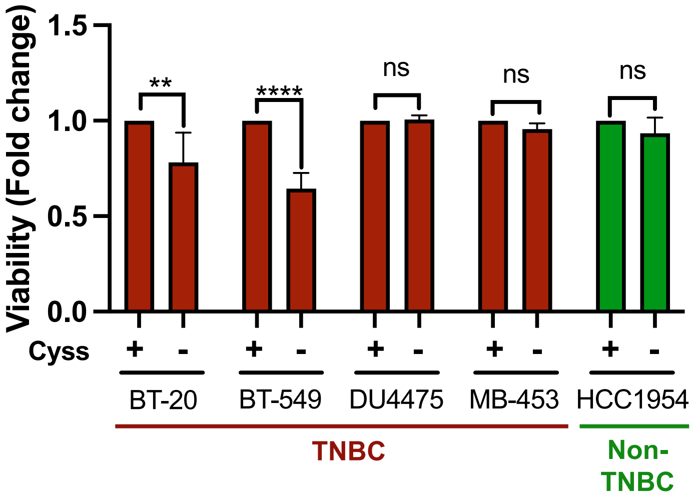

Fig. S1. Cell survival of the indicated TNBC and non-TNBC cell lines was measured after 24 h of Cyss deprivation using a cell viability assay. Viability values were calculated for each cell line as fold change relative to cells growing in complete medium. ± Cyss conditions were compared using the two-tailed unpaired t-test. An F test was used to compare variance.

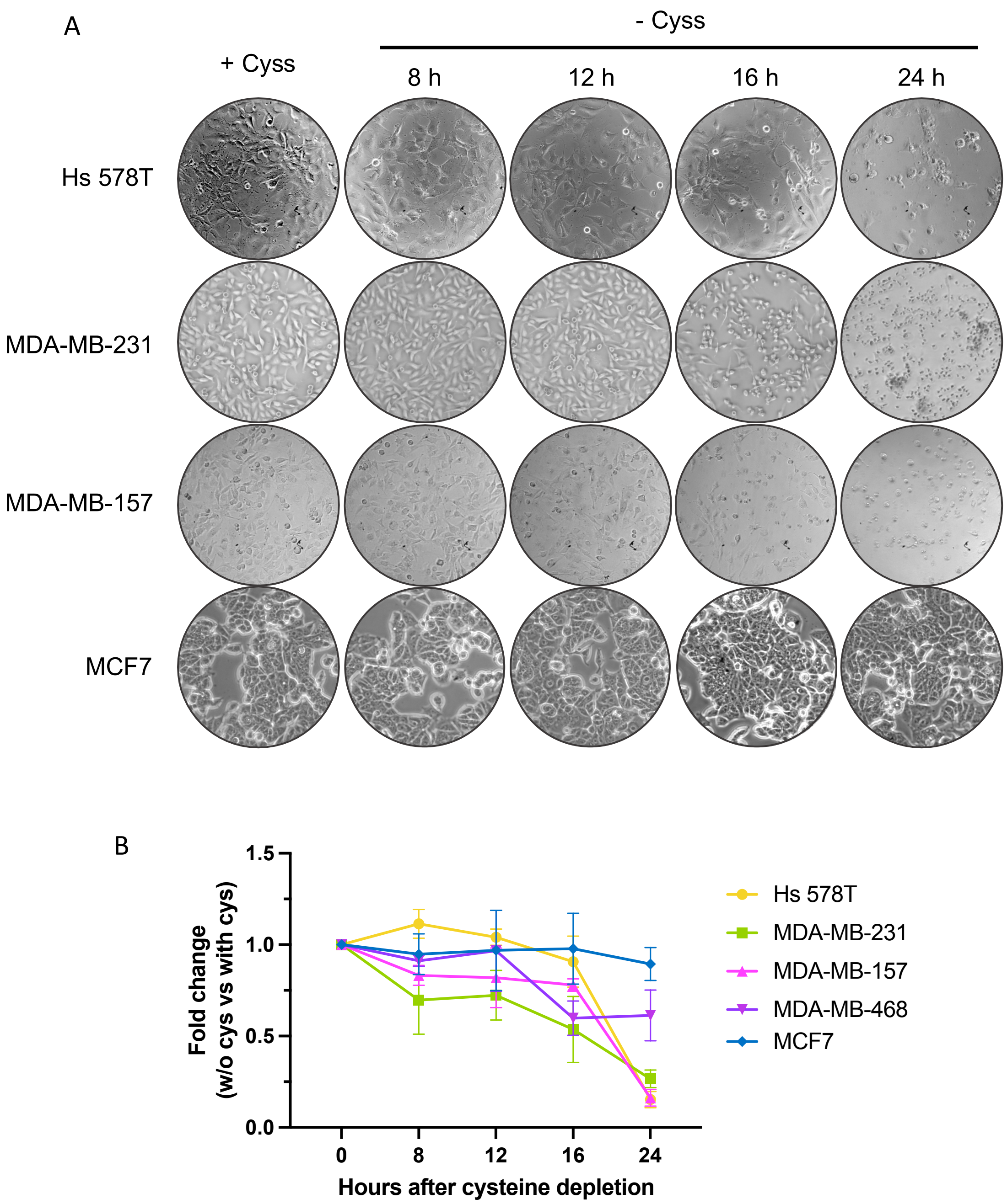

Fig. S2. A Bright-field microscopy images of TNBC (Hs 578T, MDA-MB-231, MDA-MB-157, and MDA-MB-468) and non-TNBC (MCF7) cell lines. **B** Cell survival of the indicated TNBC and non-TNBC (MCF7) cell lines was measured at the indicated time points following Cyss deprivation using a cell viability assay. Viability values were calculated for each cell line as fold change relative to cells growing in complete medium.

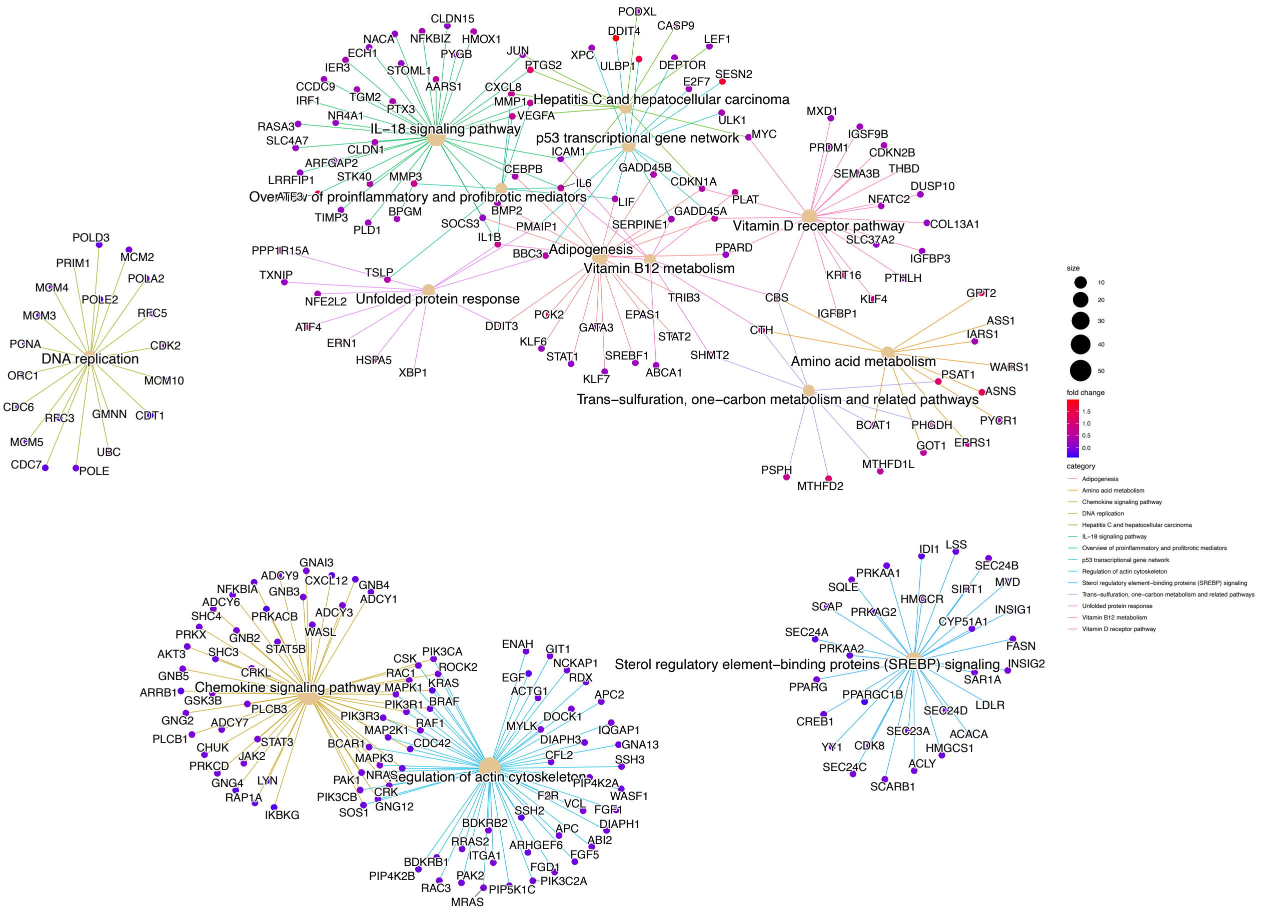

Fig. S3. Network plot depicting the linkages of genes and modulated pathways (see Fig. 2C, except those shown in Fig. 2D) that emerged after Cyss depletion.

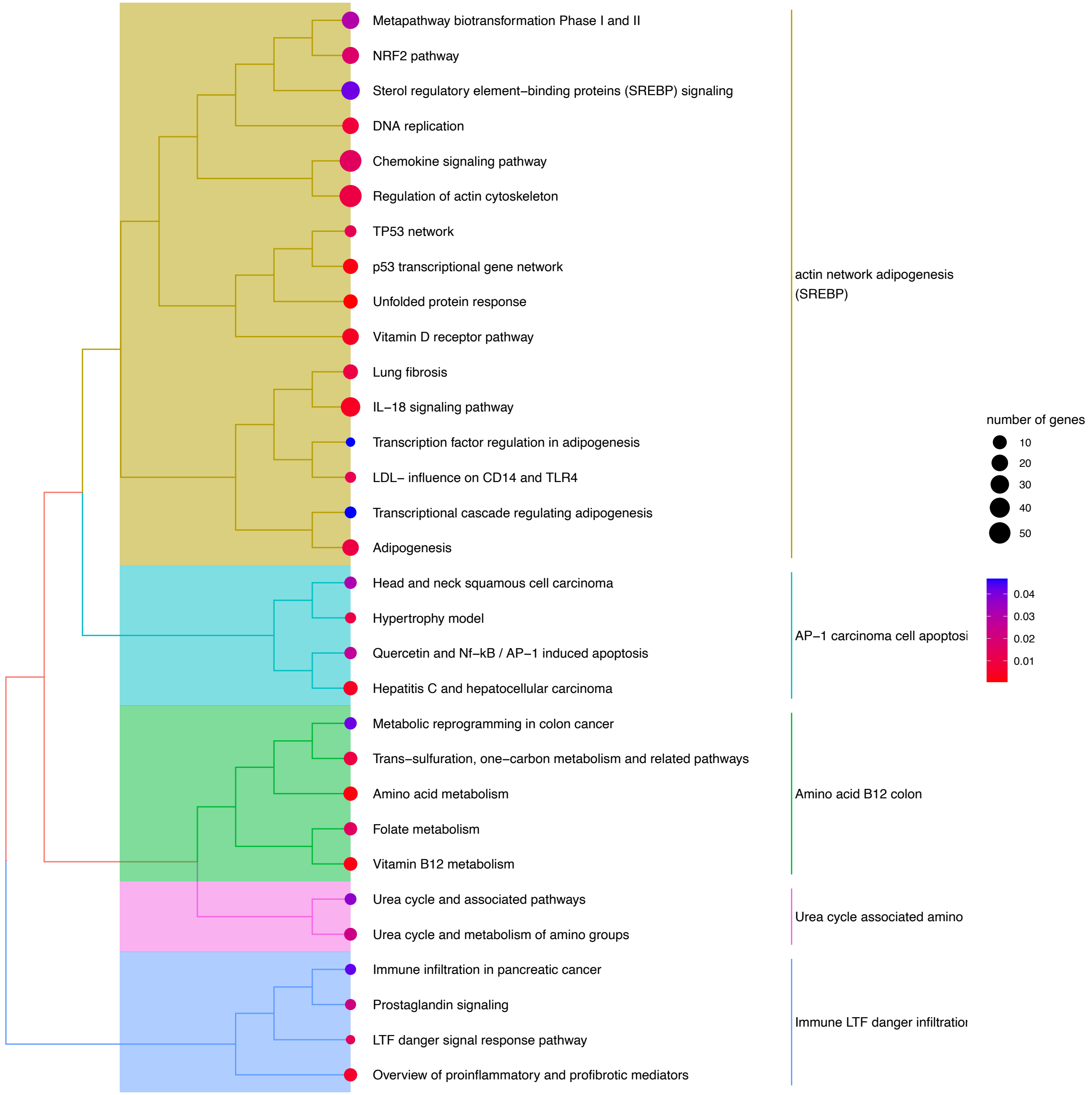

Fig. S4. Hierarchical clustering of modulated pathways based on the pairwise Jaccard similarity index, using Ward.D as the agglomeration method of average similarities between groups.

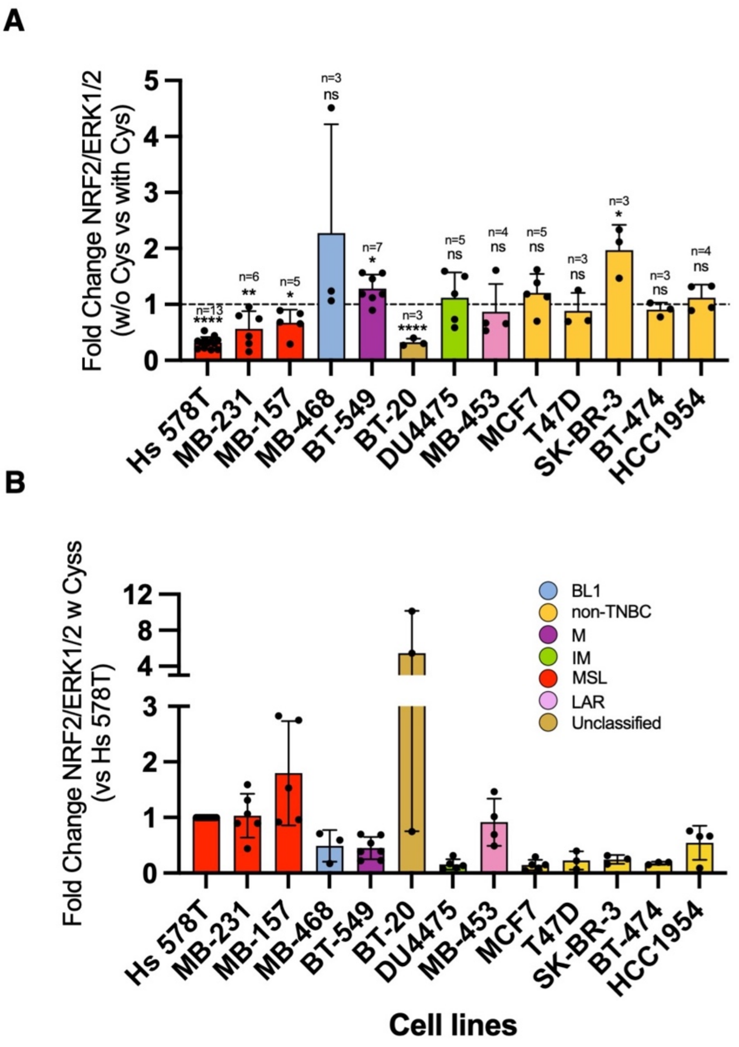

Fig. S5. Free Cyss induce NRF2 expression and activity in TNBC cell lines of the mesenchymal stem-like subtype. Representation of the NRF2 expression values in Fig. 3B as bar charts. p values (w/o Cys vs with Cys) and the number of independent experiments are indicated at the top of the columns. Values are expressed as mean ± SD. *: p≤0.05; **: p≤0.01; ***: p≤0.001; ****: p≤0.0001. ± Cyss conditions were compared using the two-tailed unpaired t-test. An F test was used to compare variance.

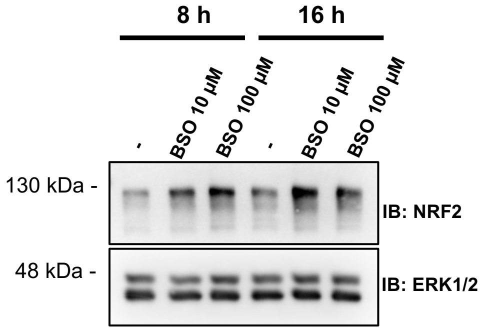

Fig. S6: In Hs 578T cells, NRF2 expression is not downregulated by interruption of biosynthetic GSH pathway. Hs 578T cells were treated with the indicated concentrations of BSO for 8 or 16 h,

and NRF2 level was analyzed by Western blot. Analysis of total ERK1/2 expression was used as loading control.

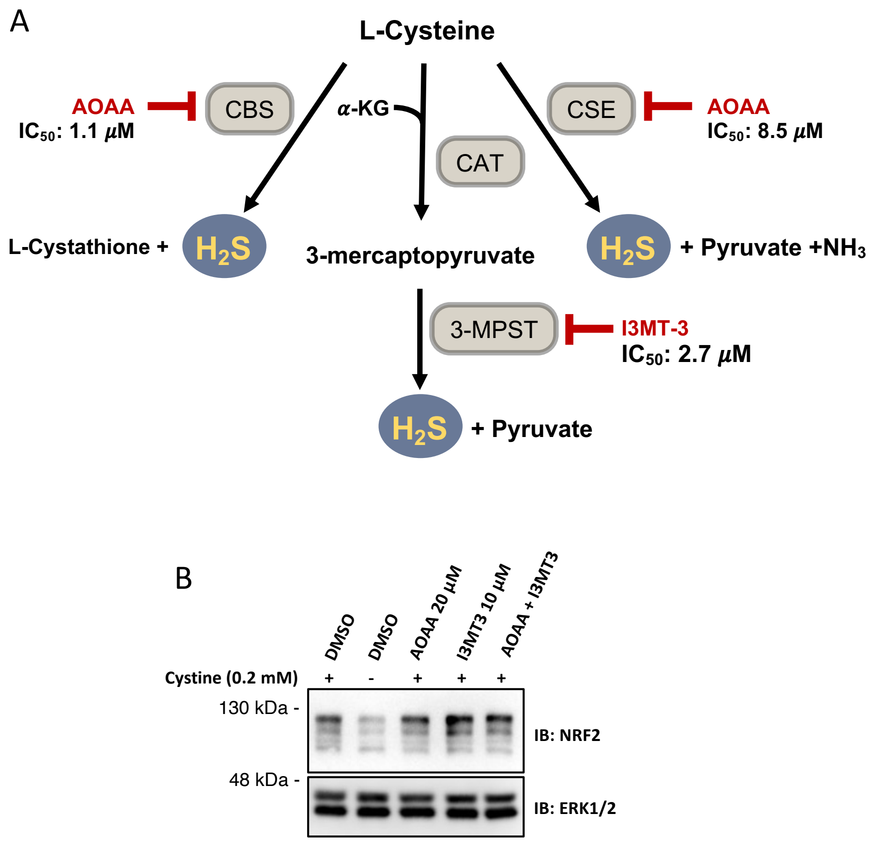

Fig. S7: In breast cancer cells, Cyss-dependent NRF2 stabilization is not dependent on intracellular H_2S. **A** Schematic representation of H_2S formation from cysteine in mammalian cells and the inhibitors used. CBS: cystathionine-β-synthase; CSE: cystathionine-γ-lyase, CAT: cysteine aminotransferase, 3-MPST: 3-mercaptopyruvate sulfurtransferase, α- KG: α-ketoglutarate, AOAA: aminooxyacetic acid. **B** Hs 578T cells were treated with pharmacological inhibitors of endogenous H_2S-producing enzymes AOAA (36)and I3MT-3 (37) for 16 h, and NRF2 level was analyzed by Western blot. As a control, an 8-hour incubation in growth media without cystine was performed. Analysis of total ERK1/2 expression was used as loading control.

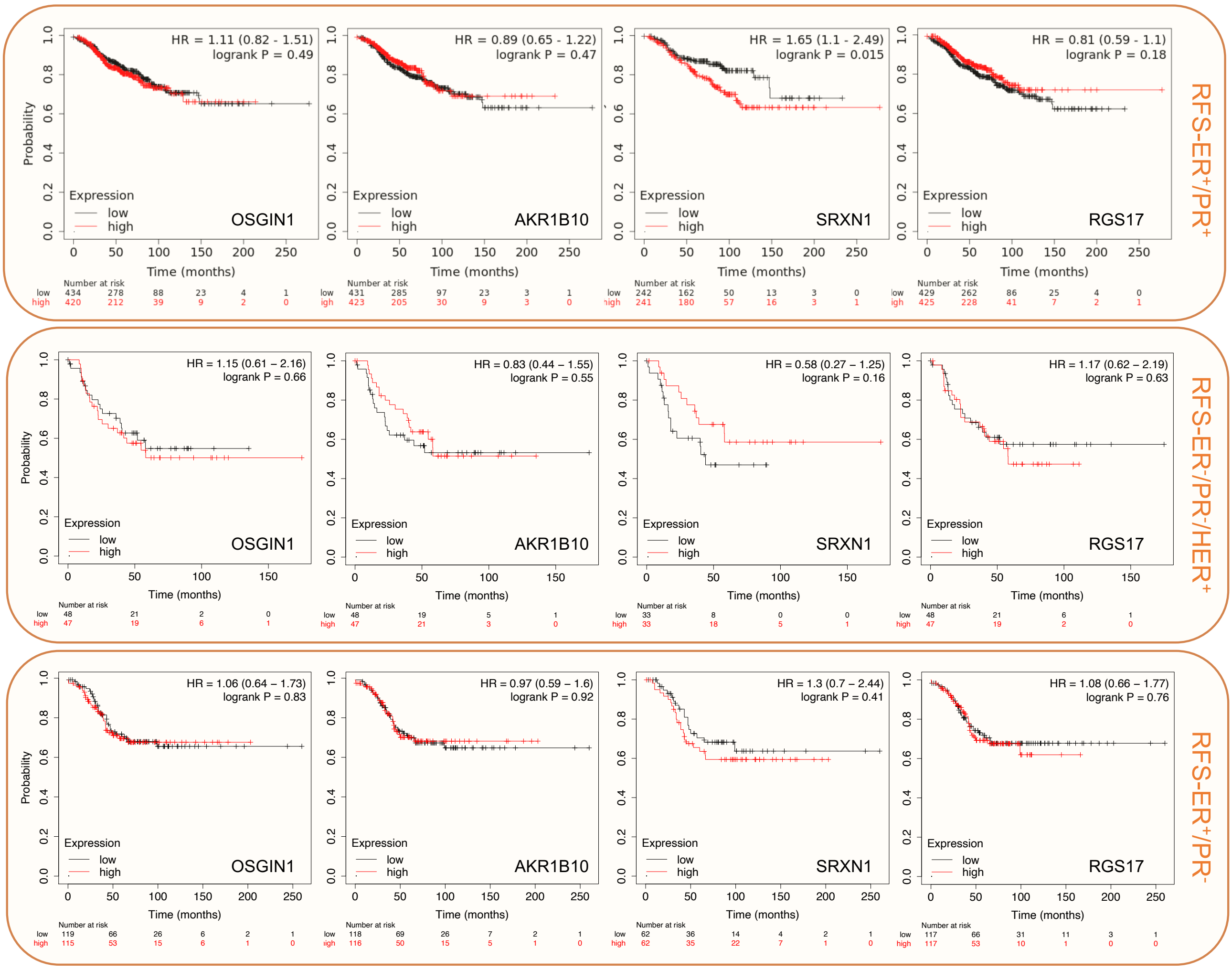

Fig. S8. Kaplan-Meier plots of the indicated genes for non-TNBC patients.

Supplementary tables

Tab. S1: List of cysteinylation sites identified in KEAP1 incubated with. The first column shows the peptide sequence where cysteinylation was found. The specific cysteinylated amino acid residues are reported in the column "Site". "Delta (ppm)" displays the mass difference between the theoretical mass of the peptide and the experimental measured mass (reported in column "Mono Mass Exp"). "RT" shows the retention time range with the most abundant MS area.

Peptide Sequence	Site	Delta (ppm)	RT	Mono Mass Exp.
CEILQSDSR	C288	3,01	4,0	1168,4888
CEILQSDSR	C288	2,17	4,0	1168,4878
LNSAECYYPER	C489	2,42	13,9	1462,5892
LNSAECYYPER	C489, A487	0,55	14,5	1494,5586
SGVGVAVTMEPCR	C613	-0,04	16,5	1423,6257
LSQQLCDVTLQVK	C77	1,58	19,1	1592,7927
LSQQLCDVTLQVK	C77	1,58	19,1	1592,7927
IFEELTLHKPTQVMPCR	C319	1,80	19,8	2160,0569
CVLHVMNGAVMYQIDSVVR	C151	1,39	24,6	2252,0605
CVLHVMNGAVMYQIDSVVR	C151	1,82	24,6	2252,0615
QEEFFNLSHCQLVTLISR	C226	-0,47	26,1	2282,0813
QEEFFNLSHCQLVTLISR	C226	-0,47	26,1	2282,0813
IYVLGGYDGHTFLDSVECYDPDTDTWSEVTR	C583	1,78	29,6	3671,5774
IYVLGGYDGHTFLDSVECYDPDTDTWSEVTR	C583	1,65	29,6	3671,5769
QEEFFNLSHCQLVTLISR	C226	-0,47	30,2	2297,0630

Tab. S2: Cell lines used in the study. HCC1954 were purchased from ATCC (Rockefeller, MD, USA), BT474, MDA-MB-453 all the other TNBC cell lines were kindly provided by Prof. S. Pece, IEO, Milan. All the cell lines were tested for mycoplasma contamination by using MycoAlert Mycoplasma Detection Kit (Lonza, Basel, Switzerland).

Cell line	TNBC subtype	Breast cancer subtype	Molecular subtype	Medium
TNBC				
BT-20	Uclassified	Basal A	Basal	MEM + 0.1mM NEAA + 1mM NaPyr + 10% FBS + 2mM L-Glut
MDA-MB-468	Basal-like 1 (BL1)	Basal A	Basal	RPMI 1640 + 10% FBS + 2mM L-Glut
DU4475	Immunomodulatory (IM)	Basal A	Basal	RPMI 1640 + 20% FBS + 2mM L-Glut + 1mM NaPyr +10mM HEPES + 2.5 g/L D-Glucose
MDA-MB-157	Mesenchymal stem-like (MSL)	Basal B	Claudin low	DMEM/F12 + 10%FBS + 2mM L-Glu
MDA-MB-231	Mesenchymal stem-like (MSL)	Basal B	Claudin low	DMEM + 10%FBS + 2mM L-Glu
BT-549	Mesenchymal-like (M)	Basal B	Claudin low	RPMI 1640 + 10% FBS + 2mM L-Glut + 0.023U/ml human insulin
Hs 578T	Mesenchymal stem-like (MSL)	Basal B	Claudin low	DMEM + 10%FBS + 2mM L-Glu + 0.01 mg/ml human insulin
MDA-MB-453	Luminal Androgen Receptor (LAR)	Luminal	Luminar AR	DMEM + 10%FBS + 2mM L-Glu
non-TNBC				
HCC1954		HER2 positive	Basal A Her^+ ER^- PR^-	
BT-474		Luminal B	luminal Her^+ ER^+ PR^+	DMEM/F12 + 10% FCS + 2mM L-Glu
MCF7		Luminal A	luminal Her^- ER^+ PR^+	RPMI 1640 + 10% FBS + 2mM L-Glut + 0.01 mg/ml human insulin
SK-BR3		HER2 positive	luminal Her^+ ER^- PR^-	DMEM + 10%FBS + 2mM L-Glu
T-47D		Luminal A	luminal Her^- ER^+ PR^+	DMEM + 10%FBS + 2mM L-Glu

Tab. S3: Primers for RT-qPCR used in the study.

Gene		5'-3' oligonucleotide sequence
GCLM	For	GGAACCTGCTGAACTGGGG
GCLM	Rev	CCCTGACCAAATCTGGGTTGA
GSR	For	GCCTTCACGAGTGATCCCAA
GSR	Rev	CTGCACCAACAATGACGCTG
NQO1	For	CGCAGACCTTGTGATATTCCAG
NQO1	Rev	CGTTTCTTCCATCCTTCCAGG
MGST1	For	TCGTGACAAAGCAAATTGTCTGG
MGST1	Rev	CCATTACCTGGGTGAGGTCAA
OSGIN1	For	CTCCAGAAAGGACCACCTCG
OSGIN1	Rev	TAGGGTGTGTAGCCGGAGAG
AKR1B10	For	AAACTGGAGGGCCTGTAACG
AKR1B10	Rev	ACAGCACCTCGATTCTCGTC

Tab. S4: Antibodies used in the study.

Antigen	Antibody	Host/clonality	Cat #
**NRF2**	NRF2	Rabbit polyclonal	#16396-1-AP – ProteinTech
**SLC7A11**	SLC7A11 Antibody (711589)	Rabbit polyclonal	# 711589 Thermo Fisher Scientific
**KEAP1**	KEAP1 (OTI1B4- formerly 1B4)	Mouse monoclonal	#TA502059 – Origene
**NQO1**	NQO1 antibody (A180)	Mouse monoclonal	#sc-32793 – Santa Cruz Biotechnology
**ERK1/2**	p44/42 MAPK (Erk 1/2)	Rabbit polyclonal	#9102S – Cell Signaling
**β-ACTIN**	Anti-β-actin (AC-15)	Mouse monoclonal	#A5441 – Sigma-Aldrich
**IgG RABBIT**	Peroxidase-conjugated AffinityPure Goat Anti-Rabbit IgG (H+L)	Goat polyclonal	#111-035-003 – Jackson Immuno Research
**IgG MOUSE**	Peroxidase-conjugated AffinityPure Goat Anti-Mouse IgG (H+L)	Goat polyclonal	#115-035-003 – Jackson Immuno Research

Supplementary methods.

Plasmid construction

Lentiviral plasmid for NRF2-ΔN89 expression was obtained by amplifying human NRF2 ORF from codon 90 (Q) to the STOP codon with the following oligonucleotides: 5'-GAGAGGATCCACCATGCAGCACATCCAGTCAGAA-3' and 5'-GAGAGTCGACCTAGTTTTTCTTAACATCTGGC-3'. Lentiviral plasmids for Myc-DDK-tagged

AKR1B10 (NM_020299) and SRXN1 (NM_080725) expression were obtained by PCR amplification of the human ORFs from plasmid RC203177 and RC207654 (ORIGENE, Rockville, MD, USA), respectively, with the following oligosnucletides: 5’-GGAATTCGTCGACTGGATC-3’ and 5’-GAGAGTCGACGTTAAACCTTATCGTCG-3’. Lentiviral plasmid for OSGIN1 expression was obtained by PCR amplification of the human ORF (NM_182981) with the following oligonucleotides: 5’-GAGAAGATCTCCACCATGAGCTCCTCCAGAAAGG-3’ and 5’-GAGAGTCGACTTAGGGTGGCTTCCTGGTC-3’. Lentiviral plasmid for DDK-tagged RGS17 (NM_012419.5) expression was obtained by PCR amplification of the human ORFs by nested RT-PCR from Hs 578T RNA with the following oligonucleotides: 5’-CACAGTAGCCTGAGGAATGC-3’, 5’- GAGAAGATCTACCATGCGAAAAAGGCAGCAGTC-3’ and 5’-GAGAGTCGACTTACTTGTCGTCATCGTCTTTGTAGTCGCCAGATTCAGAAGAAGAGCCAG -3’. All the amplicons were inserted BamHI-SalI in pCCLsin.hPGK.GFP.pre replacing the GFP ORF. The plasmid pCCLsin.hPGK.GFP.pre was used as negative control expressing GFP in lentiviral transduction experiments.
